# Educational inequalities in obesity: a multilevel analysis of survey data from cities in Latin America

**DOI:** 10.1017/S1368980021002457

**Published:** 2022-07

**Authors:** Mónica Mazariegos, Amy H Auchincloss, Ariela Braverman-Bronstein, María F Kroker-Lobos, Manuel Ramírez-Zea, Philipp Hessel, J Jaime Miranda, Carolina Pérez-Ferrer

**Affiliations:** 1 INCAP Research Center for the Prevention of Chronic Diseases (CIIPEC), Institute of Nutrition of Central America and Panama (INCAP), Guatemala City, Guatemala; 2 Department of Epidemiology and Biostatics, Urban Health Collaborative, Drexel Dornsife School of Public Health, Philadelphia, PA, USA; 3 Universidad de los Andes, Alberto Lleras Camargo School of Government, Bogotá, Colombia; 4 CRONICAS Centre of Excellence in Chronic Diseases, Universidad Peruana Cayetano Heredia, Lima, Peru; 5 CONACYT – National Institute of Public Health (INSP), Av. Universidad 655 Col. Santa María Ahuacatitlán, Cuernavaca, 62100, Mexico

**Keywords:** Inequities, Obesity, Education, Socio-economic factors, Latin America

## Abstract

**Objective:**

Using newly harmonised individual-level data on health and socio-economic environments in Latin American cities (from the Salud Urbana en América Latina (SALURBAL) study), we assessed the association between obesity and education levels and explored potential effect modification of this association by city-level socio-economic development.

**Design:**

This cross-sectional study used survey data collected between 2002 and 2017. Absolute and relative educational inequalities in obesity (BMI ≥ 30 kg/m^2^, derived from measured weight and height) were calculated first. Then, a two-level mixed-effects logistic regression was run to test for effect modification of the education–obesity association by city-level socio-economic development. All analyses were stratified by sex.

**Setting:**

One hundred seventy-six Latin American cities within eight countries (Brazil, Chile, Colombia, Costa Rica, El Salvador, Guatemala, Mexico and Peru).

**Participants:**

53 186 adults aged >18 years old.

**Results:**

Among women, 25 % were living with obesity and obesity was negatively associated with educational level (higher education–lower obesity) and this pattern was consistent across city-level socio-economic development. Among men, 18 % were living with obesity and there was a positive association between education and obesity (higher education–higher obesity) for men living in cities with lower levels of development, whereas for those living in cities with higher levels of development, the pattern was inverted and university education was protective of obesity.

**Conclusions:**

Among women, education was protective of obesity regardless, whereas among men, it was only protective in cities with higher levels of development. These divergent results suggest the need for sex- and city-specific interventions to reduce obesity prevalence and inequalities.

Despite recent economic improvements, Latin America has one of the highest levels of wealth inequality in the world^(1)^ and health inequalities follow wealth inequality^(2)^. Research on health inequalities in Latin America has primarily focused on children’s health outcomes and access to maternal health services^(2,3)^. Wealth-related inequalities in obesity have been less studied despite the fact that chronic diseases related to obesity are rapidly rising in the region and current evidence suggests they are socially patterned^(4,5)^.

Recent evidence has highlighted sex differences in the prevalence of obesity by socio-economic status.

The prevalence of obesity tends to be highest among women with lower socio-economic position in Latin America, whereas among men, results were inconsistent and depended on the measure of socio-economic position used^(6–8)^. Further, it is possible that – in addition to sex differences – the effect of socio-economic position on obesity differs by country-level socio-economic development^(9)^.

Latin American countries and cities have different levels of economic and social development and are at different stages of the epidemiological and nutrition transition^(10)^; therefore, it is likely that the stage of obesity transition varies between and within countries^(11)^. As a country’s economy grows, the prevalence of obesity increases and the burden of obesity tends to shift from advantaged to disadvantaged populations^(10)^. This phenomenon likely occurs because increases in socio-economic position serve to protect against the obesogenic effects of economic development. For example, education has the potential to affect health ‘twice over’ by affecting a person’s exposure and receptivity to health education messages promoting healthier behaviours and indirectly by offering employment and income opportunities which affect health in many ways including expanding opportunities for health-promoting behaviours^(12–14)^. Furthermore, education is associated with changing cultural norms related to thinness and attractiveness^(5,12)^ which may help to explain the sex differences in social patterning of obesity and how this pattern varies by economic development.

While most prior work has focused on country-level socio-economic development and its association with the social patterning of health outcomes, city-level socio-economic development varies widely within countries^(11)^. One of the sustainable development goals (goal 10)^(15)^ is to reduce inequalities between and within countries and cities. A critical unmet step to move towards this goal in relation to obesity is to monitor educational inequalities in obesity at the city level.

Our objective was to estimate educational inequalities in obesity and examine whether a city-level economic development index modifies the social gradient in obesity. The hypotheses are that those with lower education will have higher prevalence of obesity, and the association will be most apparent in cities with higher socio-economic development. Because most work has found heterogeneity in obesity by sex^(16,17)^, all analyses were stratified by sex. We hypothesised that the gap between lower and higher education will increase as city-level socio-economic development increases and this will be stronger for women than for men.

## Methods

### Study setting

This cross-sectional study was conducted as a part of the SALURBAL (Salud Urbana en América Latina) research project^(18)^. SALURBAL has compiled and harmonised health, social and physical environment data on all cities with a population above 100 000 (*n* 371 cities) in eleven Latin American countries (Argentina, Brazil, Chile, Colombia, Costa Rica, El Salvador, Guatemala, Mexico, Nicaragua Panama and Peru). For this study, we used survey data from 176 cities in eight countries (all except Argentina, Nicaragua and Panama) collected between 2002 and 2017 which included 53 186 adults aged >18 years old. Cities that had a small number of survey participants (*n* 158), had self-reported weight and height (*n* 36) or did not include data for the socio-economic development index (*n* 1) were not included in the present analysis. See Supplemental Table 1 for more details on the number of cities and participants considered for each country.

### Survey data

For Brazil (2013), Chile (2010), Colombia (2007), El Salvador (2014), Mexico (2016) and Peru (2017), we used data from the nationally representative cross-sectional health surveys. For Guatemala (2002) and Costa Rica (2005), we used data from the Central America Diabetes Initiative Survey CAMDI^(19)^ a probabilistic sample of the noninstitutionalised population. Survey details are provided in Supplemental Table 1.

The main outcome variable was individual-level obesity defined as a BMI ≥ 30 kg/m^2^ using the WHO cut-off points^(20)^. Weight and height were objectively measured in all surveys following standard procedures. The main exposure variable was individual-level educational attainment which was harmonised across countries/surveys and classified into less than primary, primary, secondary, university or higher. Individual-level covariates included age (continuous variable) and sex, since both these variables are associated with educational attainment and obesity prevalence. Data from all surveys were restricted to men and non-pregnant women aged >18 years old.

### Area-level data

Available census data were compiled from each country/city that matched the survey year as closely as possible (see online Supplemental Table 1). Five variables were selected to represent city-level socio-economic development: (1) Water access (% households with access to piped water); (2) Sanitation (% households with access to a municipal sewage network); (3) Durable walls (% of dwellings with exterior walls mostly made of brick, stone, concrete, cement and/or similar materials); (4) Overcrowding (% households with more than three people per room) and (5) Education (% population with at least completed primary education among those aged 25 years or above). We created a city-level socio-economic development index to proxy economic development^(21)^ by summing the standardised *Z*-scores of the five variables (after reversing overcrowding). Then, this index was categorised in tertiles to represent low, middle and high socio-economic development.

See Supplemental Table 2 and Supplemental Fig. 1 for more detailed information on the distribution of the socio-economic development index in Latin American cities by country.

### Statistical analysis

First, we estimated the city-level age-standardised proportion of obesity and 95 % CI using the WHO standard population^(22)^. To assess educational inequalities in obesity, we present the distribution of obesity by categories of education. We also computed absolute difference in obesity for higher *v*. lower education categories (‘lowest-highest educational level difference’) and the Relative Index of Inequality (RII)^(23)^. The RII is a regression-based measure that considers the whole population education distribution rather than only the two extreme education categories. To estimate the RII, education level was transformed onto a scale from 0 (highest level of education) to 1 (lowest level of education) and weighted to reflect the share of the population at each educational level by calculating the midpoint of the proportion in the population in each category. The RII thus takes into account the relative size of each socio-economic group in calculating overall inequality. To obtain the RII, obesity was regressed on the new education variable in generalised linear models adjusted for age, with a logarithmic link function to calculate the RII. The RII is interpreted as the prevalence ratio between the two ends of the educational hierarchy – obesity proportion at the bottom divided by obesity proportion at the top. If there is no inequality, RII takes the value of 1. Values >1 indicate a higher concentration of obesity among the disadvantaged, and values <1 indicate a higher concentration of obesity among the advantaged population.

Second, we used a two-level mixed-effects logistic regression model to examine how educational differences in the odds of obesity are influenced by country differences, and city-level socio-economic development. The unit of analysis was individuals. A random intercept was used for city and random coefficient for education. We defined four models *a priori*: empty model, model 1 (unadjusted), model 2 (age adjusted + country-fixed effects) and model 3 (model 2 + city-level socio-economic development). We included country as a fixed effect to control for any unmeasured country factors such as differences in education systems across countries. We next evaluated potential effect measure modification by examining the association between education and obesity stratified by city-level socio-economic development.

All models were stratified by sex because of well-established evidence of heterogeneity in educational effects on obesity by sex^(16,17)^. We present results from unweighted analyses due to minimal differences from scaled weighted and unweighted estimates and because of the complexity of using weights when fitting multilevel models^(24)^. All analyses were conducted in the statistical software package Stata version 15.0. We used the xtmelogit command for the multilevel modelling analysis.

## Results

Chile, Colombia and Mexico have the highest scores for city-level socio-economic development, followed by Brazil, Peru, Costa Rica, Guatemala and El Salvador (see online Supplemental Fig. 1).

The percentage of adults with high education (University or higher) ranged from 2–12 % (cities of Guatemala, El Salvador, Mexico, Colombia, Chile and Peru) to 17–29 % (cities of Costa Rica and Brazil).

In general, women had higher proportion of obesity compared with men (24·7 % *v*. 18·4 %, respectively, see Table 1 and see online Supplementary Figs. 2 and 3). In women, obesity was higher among those in the lower education level groups (except for Costa Rica). Among women, the largest percentage points (pp) gap in obesity between lowest-and-highest education level was in Chile (20·0 pp) (Table 1). Using the RII, obesity was between 20 % and 120 % more prevalent among the least educated women compared with the most educated women in the population (Costa Rica; RII: 1·2, 95 % CI 0·8, 1·8 and Guatemala; RII: 2·2, 95 % CI 0·8, 6·5). Among men, education disparities in obesity were inconsistent. There was no pattern by education in four countries, obesity was higher among lower educated groups in Costa Rica (10·7 pp) and Mexico, (8·9 pp) and higher among higher educated groups in Guatemala (−17·7 pp), and El Salvador (−10·2 pp). Obesity was between 10 % and 50 % less prevalent among the least educated compared with the most educated men in the population (Costa Rica; RII: 0·9, 95 % CI 0·4, 1·8 and El Salvador; RII: 0·5 95 % CI 0·2, 1·0) (Table 1).

**Table 1:** Age-standardised obesity proportion by educational level in 176 Latin American cities by country‡

			By country															
	Pooled sample		Brazil		Chile		Colombia		Costa Rica		El Salvador		Guatemala		Mexico		Peru	
Women (*n*)	31 708		14 846		1490		3449		741		998		715		2723		6746	
All	24·7	24·2, 25·2	21·9	21·2, 22·6	27·8	25·6, 30·3	16·5	15·4, 17·8	24·9	22·1, 28·1	33·2	30·4, 36·1	24·5	21·7, 27·5	40·6	38·8, 42·4	28·3	27·2, 29·4
Educational level																		
Less than primary	29·9	28·0, 31·8	28·4	25·9, 30·9	38·6	24·6, 54·8	19·8	16·8, 23·3	9·7	2·0, 35·7	32·1	26·8, 37·9	27·1	22·3, 32·5	47·8	40·7, 54·9	36·7	31·9, 41·9
Primary	29·3	28·3, 30·3	25·7	24·2, 27·2	34·1	29·6, 38·9	17·2	15·2, 19·3	27·6	22·6, 33·3	36·4	31·8, 41·1	29·0	23·8, 34·8	41·8	39·3, 44·3	34·6	31·8, 37·5
Secondary	22·3	21·1, 23·1	20·2	19·1, 21·3	24·9	21·7, 28·4	15·9	13·1, 19·3	26·4	21·3, 32·4	32·3	26·3, 39·1	24·2	13·2, 40·2	35·4	31·1, 40·1	26·4	24·8. 28·0
University or higher	19·3	14·8, 24·8	18·2	12·7, 25·3	17·7	12·1, 25·1	14·4	9·9, 20·6	25·4	19·4, 32·6	15·7	7·7. 29·3	–	−*	31·5	24·4, 39·6	22·9	19·9, 26·2
Lowest-highest educational level difference (pp)	10·6	10·4, 10·8	10·2	10·1, 10·3	20·9	20·6, 21·2	5·4	5·2, 5·6	−15·7	−15·3, −16·1	16·4	16·3, 16·5	2·9	2·7, 3·1†	16·3	16·2, 16·4	13·8	13·7, 13·9
Relative index of inequality§	1·7	1·6, 1·8	1·9	1·7, 2·1	1·8	1·4, 2·5	1·4	1·1, 1·9	1·2	0·8, 1·8	1·2	0·7, 1·7	2·2	0·8, 6·5	1·6	1·3, 1·9	1·5	1·3, 1·7
Men (*n*)	21 478		10 570		975		2300		423		497		323		1343		5047	
All	18·4	17·8, 18·9	17·3	16·6, 18·0	22·2	19·7, 25·0	11·5	10·3, 12·9	20·5	16·9, 24·6	24·4	20·8, 28·4	15·6	12·0, 20·2	29·5	27·1, 32·1	21·0	19·9, 22·1
Educational level																		
Less than primary	16·2	14·6, 17·8	16·0	14·1, 18·1	11·9	4·6, 27·2	13·6	8·9, 20·1	27·9	18·4, 39·9	16·4	10·7, 24·6	17·1	9·9, 27·6	34·8	25·5, 45·4	13·5	10·4, 17·3
Primary	18·0	17·1, 19·1	15·8	14·4, 17·3	24·4	19·6, 29·9	12·5	10·5, 14·8	17·7	11·7, 25·9	26·2	20·6, 32·7	16·6	11·2, 23·9	28·7	25·4, 32·2	19·6	17·0. 22·4
Secondary	19·9	19·0, 20·8	19·1	17·8, 20·4	21·8	18·2, 25·8	10·5	8·3, 13·1	23·5	17·6, 30·0	27·9	20·7, 36·4	11·5	5·3, 23·4	31·5	26·0, 37·6	22·4	20·9, 24·1
University or higher	17·7	16·4, 18·9	16·5	15·0, 18·1	14·1	8·4, 22·5	14·2	9·4, 20·9	17·2	12·0, 23·8	26·6	14·9, 42·8	34·8	12·2, 67·4	25·9	18·3, 35·3	22·3	19·4, 25·6
Lowest-highest educational level difference (pp)	−1·5	−1·4, −1·6	−0·5	−0·4, −0·6	−2·2	−1·8, −2·5	−0·6	−0·4, −0·8	10·7	10·2, 11·2	−10·2	9·8, 10·6	−17·7	−17·2, −18·2	8·9	8·6, 9·2	−8·8	−8·7, −8·9
Relative index of inequality§	0·8	0·7, 0·9	0·8	0·7, 0·9	1·3	0·9, 2·1	1·0	0·67. 1·6	0·9	0·4, 1·8	0·5	0·2, 1·0	0·7	0·2, 2·6	1·1	0·8, 1·6	0·7	0·5, 0·8

* There is no available data for women with university or higher educational level.

† Comparison made between less than primary and secondary education.

‡ Number of cities with ≥100 000 inhabitants (according to 2010 census estimates) in each SALURBAL country: Brazil: 27; Chile: 29; Colombia: 33; Costa Rica and Guatemala: 1; El Salvador: 3; Mexico: 59 and Peru: 23. These cities are a collection of municipalities and were identified using various databases and a practical and systematic protocol.

§ Relative index of inequality (RII) is a measure of inequity. If there is no inequality, RII takes the value of 1. Values >1 indicate a concentration of obesity among the disadvantaged, and values <1 indicate a concentration of obesity among the advantaged population.

All data are expressed as % (95 % CI); RII are expressed as prevalence ratio and 95 % CI.

**Fig. 1:**
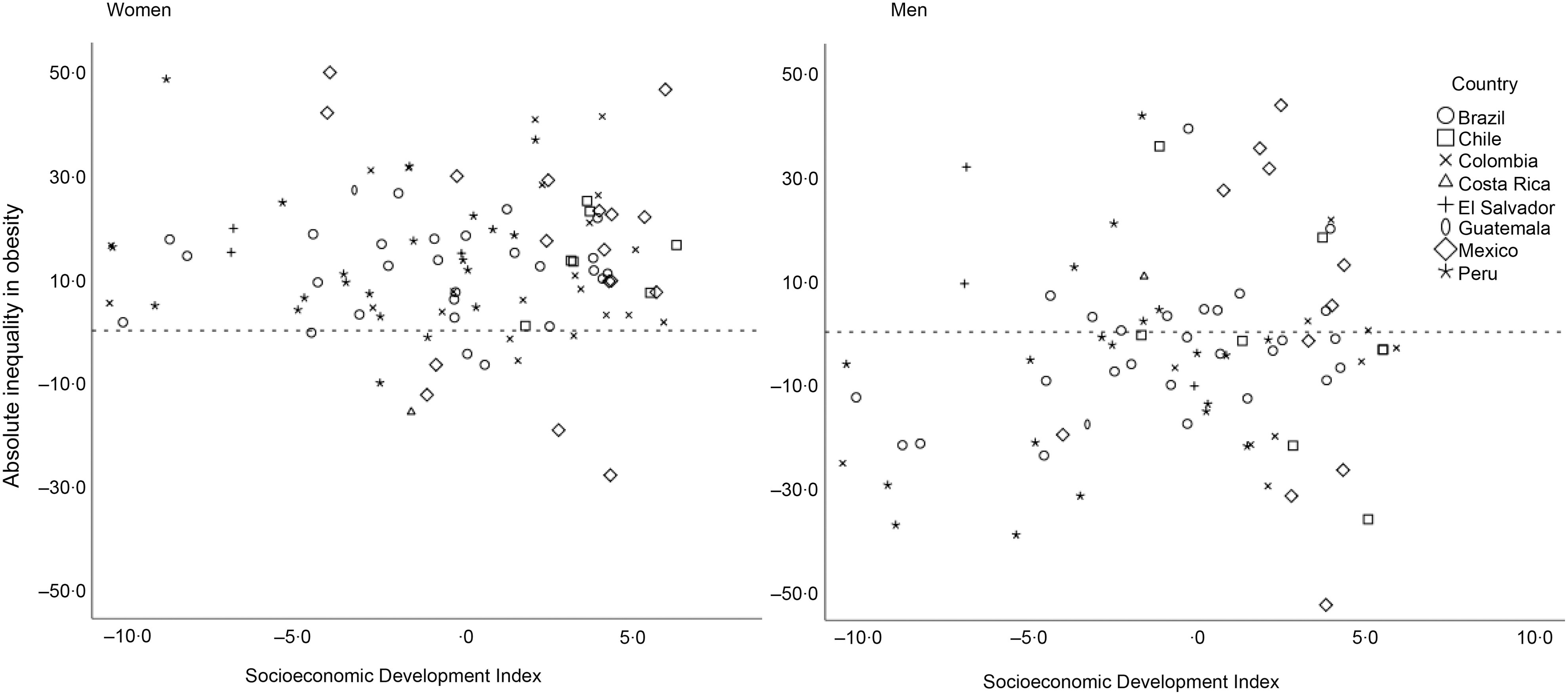
Absolute inequality in obesity by city-level socio-economic development index in women and men

In descriptive analyses (Table 2), we found that among women, absolute inequalities in obesity between lowest-and-highest educational levels widened slightly as city’s socio-economic development score improved. Among men living in cities with the lowest socio-economic development scores, obesity was highest among more educated men (−7·8 pp). As city socio-economic development scores improved, the gap narrowed (−1·5 pp) and then the social gradient reversed so that obesity became more prevalent among the least educated men (3·2 pp). Relative inequalities measured with the RII show a similar pattern across the categories of city socio-economic development. Figure 1 highlights the city variation in each country and shows the absolute inequality in obesity by city-level socio-economic development index in women and men. Among women, there is a higher proportion of cities with an inverse association between obesity and education compared with men.

**Table 2:** Age-standardised obesity proportion by educational level‡ classified by city-level socio-economic development index and stratified by sex

	Socio-economic development index§					
	Low tertile		Medium tertile		High tertile	
Women (*n*)	10 437		11 944		8926	
All	26·0	25·2, 26·8	27·1	26·3, 27·9	25·2	24·3, 26·1†
Educational level						
Less than primary	31·0	29·2, 32·9	35·5	33·4, 37·5	33·4	31·3, 35·8
Primary	28·7	27·1, 30·4	33·2	31·5, 34·9	30·9	29·3, 32·7
Secondary	22·9	21·7, 24·2	22·9	21·8, 24·1	19·1	17·7, 20·5
University or higher	21·0	18·8, 23·4	18·9	17·2. 20·8	16·5	14·5, 18·7
Lowest-highest educational level difference (pp)	10·0	9·9, 10·1	16·6	16·5, 16·7*	16·9	16·8, 17·0*,†
Relative index of inequality‖	1·4	1·2, 1·6	1·8	1·6, 2·0	2·1	1·8, 2·4
Men (*n*)	7015		8467		5687	
All	19·8	18·9, 20·8	20·8	20·0, 21·2	17·7	16·8, 18·7*,†
Educational level						
Less than primary	18·0	16·0, 20·2	20·2	18·1, 22·5	18·3	16·0, 20·9
Primary	17·9	16·3, 19·7	20·6	18·8, 22·5	18·7	16·9, 20·5
Secondary	20·2	18·8, 21·6	20·9	19·7, 22·2	17·8	16·2, 19·5
University	25·8	22·9, 28·8	21·7	19·6, 23·8	15·1	12·8, 17·7
Lowest-highest educational level difference (pp)	−7·8	−7·7, −7·9	−1·5	−1·4, −1·6*	3·2	3·1, 3·3*,†
Relative index of inequality‖	0·6	0·5, 0·8	0·8	0·7, 0·9	1·1	0·8, 1·3

All data are expressed as % (95 % CI); RII are expressed as prevalence ratio and 95 % CI.

* *P* < 0·05 *v*. low tertile.

† *P* < 0·05 *v*. medium tertile.

‡ Data come from surveys collected in 177 cities.

§ Socio-economic development index is the sum of the standardised *Z*-scores of the five variables (reversing overcrowding): water access, sanitation, durable walls, overcrowding and contextual education.

‖ Relative index of inequality (RII). If there is no inequality, RII takes the value of 1. Values >1 indicate a concentration of obesity among the disadvantaged, and values <1 indicate a concentration of obesity among the advantaged population.

Table 3 shows the odds of obesity adjusted for age, sex, country, before and after adjusting for city socio-economic development index. Individual-level education was inversely associated with obesity in women but positively associated in men. City-level socio-economic development was not directly associated with obesity among women or men.

**Table 3:** Odds ratios (OR) and 95 % CI for obesity according to education level and socio-economic development index, stratified by sex

	Model 1†		Model 2‡		Model 3§	
	OR	95 % CI	OR	95 % CI	OR	95 % CI
Women (*n* 31 708)						
Individual-level variable						
Educational level						
Less than primary	Reference		Reference		Reference	
Primary	0·87	0·81, 0·94*	1·09	1·00, 1·17*	1·08	0·99, 1·17*
Secondary	0·56	0·52, 0·60*	0·76	0·70, 0·82*	0·74	0·69, 0·81*
University or higher	0·46	0·42, 0·51*	0·58	0·53, 0·64*	0·58	0·53, 0·64*
City-level variable						
Socio-economic development index						
Low tertile					Reference	
Medium tertile					1·00	0·88, 1·14
High tertile					0·90	0·88, 1·05
Men (*n* 21 478)						
Individual-level variable						
Educational level						
Less than primary	Reference		Reference		Reference	
Primary	1·02	0·92, 1·15	1·23	1·11, 1·42*	1·23	1·09, 1·38*
Secondary	1·07	0·96, 1·18	1·39	1·23, 1·59*	1·37	1·20, 1·57*
University or higher	1·16	1·03, 1·31*	1·38	1·17, 1·62*	1·36	1·15, 1·61*
City-level variable						
Socio-economic development index						
Low tertile					Reference	
Medium tertile					1·00	0·86, 1·17
High tertile					0·86	0·71, 1·00

A random intercept was used for cities.

* *P* < 0·05.

† Model 1: Unadjusted model.

‡ Model 2: Age-adjusted and fixed effect for country.

§ Model 3: Model 2 + added city-level Socio-economic Development Index (contextual variable).

Stratified analyses revealed that the association between education and obesity varied by levels of the city social development especially among men. Among men, there was a clear positive association (higher education–higher obesity) in cities with low socio-economic development scores. The educational gradient reversed as city-level socio-economic development improved and an inverse association emerged in cities with the highest socio-economic development scores. Among women, decreases in obesity with higher education were consistent across different levels of city-level socio-economic development index (Table 4).

**Table 4.** OR (95 % CI) for obesity according to education, stratified by sex and city-level socio-economic development index

	Socio-economic development index					
	Low		Medium		High	
Women (*n* 31 708)	OR	95 % CI	OR	95 % CI	OR	95 % CI
Educational level						
Less than primary	Reference		Reference		Reference	
Primary	1·10	0·96, 1·24	1·00	0·83, 1·19	0·93	0·77, 1·12
Secondary	0·85	0·74, 0·97*	0·84	0·71, 0·99*	0·78	0·64, 0·94*
University	0·71	0·60, 0·85*	0·77	0·61, 0·97*	0·71	0·55, 0·91*
Men (*n* 21 478)						
Educational level						
Less than primary	Reference		Reference		Reference	
Primary	1·24	1·02, 1·51*	0·99	0·75, 1·29	0·96	0·71, 1·28
Secondary	1·47	1·23, 1·77*	0·89	0·68, 1·19	0·90	0·69, 1·20
University	1·89	1·51, 2·37*	0·70	0·52, 0·94*	0·51	0·36, 0·72*

All models adjusted for age and country dummies.

* *P* < 0·05.

## Discussion

### Summary

Using individual-level data for residents of 176 Latin American cities, we found that obesity was inversely associated with education among women in most cities and educational inequalities were large. Among women, we did not find evidence that educational inequalities in obesity were larger as city-level socio-economic development improved (there were no differences across levels of socio-economic development). Among men, we found evidence of a reversal of the educational gradient – from a direct association to an inverse association – as city-level socio-economic development improved.

### Women with more obesity than men

In our analysis, a higher proportion of women were living with obesity than men. This is consistent with other studies that have reported higher BMI in women than in men^(7,25)^. Women are at greater risk of obesity^(26)^ due to reproductive factors such as early age at menarche and parity that promote weight gain and adiposity through repetitive cycles across the life course^(26–28)^.

### Women – higher education, lower obesity; Men – higher education, higher obesity

Obesity was inversely associated with education among women, but among men, there was a pattern of higher obesity among the more educated. Our results are consistent with a recent analysis from Mexico, Colombia and Peru where higher education was inversely associated with BMI in women but somewhat positive in men^(7,25)^. Gender differences may be associated with different social norms about thinness and attractiveness between men and women^(29)^. For example, among men, a larger body size may be valued as a sign of physical dominance, while thinness may be valued among women especially in socially advantaged groups^(30,31)^. Furthermore, men with fewer years in education may be employed in manual occupations that require high levels of physical activity which confers protection from obesity^(32)^. Less advantaged women may be less physically active and in addition are targeted by the food industry to increase sales of energy-dense nutrient-poor foods^(33–36)^. Results from a study that explored dietary patterns in Mexico City showed that socially disadvantaged households relied more heavily on local corner stores that offer mainly cheap and poor-quality food^(37)^.

### Effect modification by city-level development

It has been proposed that the reversal of the social gradient for men occurs at higher levels of development than for women^(38)^. Our findings support this as we found evidence of a reversal of the educational gradient as city-level socio-economic development improved. A study in Argentina found that the inverse socio-economic patterning of chronic disease risk factors (i.e. lower risk factor levels in the more advantaged groups) became stronger or only emerged in more urban settings, and the effect modification of urbanisation was stronger in men than in women^(39)^. In contrast, a study in Brazil found that high education was inversely associated with obesity in women, and positively associated among men^(40)^. We speculate that more educated men living in cities with high socio-economic development may be able to handle obesogenic environments by greater adherence to healthier lifestyles (greater consumption of fruit and vegetables, less consumption of ultra-processed food and more engagement in recreational physical activities), use of nutritional information and may also have more access to healthy food^(41,42)^ and better skills to prepare healthy meals^(43,44)^ than their less-educated counterparts. All these factors show the complex way in which socio-economic development, sex and education interact to shape obesity. Understanding the drivers (including attitudes related to masculinity and moralising consumption of certain foods)^(43)^ that make high educated men living in cities with high socio-economic development less vulnerable to obesogenic environments is of great importance to address existing inequalities.

### Policy significance

Our results are relevant for public policy in several respects. First, we identify large inequalities in obesity within Latin American cities. Second, we identify that lower education is associated with obesity among women across the socio-economic development spectrum and with men in more developed cities. Hence, interventions could be directed to: (a) increasing education level and narrowing educational inequalities within cities and countries and (b) combining both population-level interventions and targeted interventions to the most vulnerable to narrow inequalities. Examples of targeted interventions to high-risk individuals (women and men with low educational level) are physician and nutritional counselling at primary health services and multi-component workplace-based interventions^(45,46)^. However, evidence shows that interventions targeting individual-level behaviour changes are less successful; hence, population-level interventions are also key^(47,48)^. Population-level interventions are more effective and equitable and include community-based strategies and policies that change the environment (e.g. food reformulation, active streets, and marketing regulations). Population level policies require individuals to use a low level of resources (cognitive, psychological, time, and material resources) and make the healthy choice the default choice^(47,48)^.

### Strengths and limitations

Our study had strengths and limitations. The strengths of the study include the use of harmonised data on 176 cities in eight countries. In addition, its multilevel analysis allowed us to analyse individual and macro-level contextual factors. Furthermore, using the socio-economic development index for cities allowed for the assessment of social development in addition to only economic development of Latin American cities^(21)^. Our study provides novel important information on how both individual and contextual factors shape the association between obesity and education. However, surveys and sample size differ by country, and number of cities differs; hence, the association between education and obesity may not reflect the state of their current stage in the epidemiological, nutrition and obesity transition. We attempted to address this by adding a country-fixed effect in models. Another limitation is that meaning of education may vary for different birth cohorts^(8,49)^. Older cohorts will be overrepresented among those classified as less educated because educational attainment has improved over the years. Furthermore, quality of education or the curriculum may have changed throughout the years or may be different across countries and cities^(49)^. Knowledge and skills learned in education may differ for older and younger generations^(49)^. However, the strengths of the use of education as a measure of socio-economic position are that it is commonly collected in health surveys^(8)^, is the most frequently used socio-economic indicator in obesity studies, and its use allows comparability with other studies. It is also less prone to recall bias and more reliable over time than other socio-economic position measures such as income^(50)^. In addition, we did not include analysis for more disaggregated data such as sub-city units and smaller neighbourhoods. City-level measurements may mask within city inequalities which are worth exploring in future research. Last, individual-level behavioural variables associated with obesity such as diet, physical activity, and sitting time are lacking, and their inclusion in upcoming studies is needed.

## Conclusion

In conclusion, given the short- and long-term health consequences of obesity, it is important to lighten the burden for more disadvantaged groups. The high proportion of obesity among those less-educated suggests the urgent need of equity-based and context-sensitive interventions at population level and policies that anticipate inequalities in obesity-related diseases and avoid widening inequities among social groups.

## Supplementary material

For supplementary material accompanying this paper visit https://doi.org/10.1017/S1368980021002457. click here to view supplementary material
