# Supplementary material for: Educational inequalities in obesity: a multilevel analysis of survey data from cities in Latin America
Source: Public Health Nutr. 2021 Jun 25;25(7):1790–8. doi: 10.1017/S1368980021002457 (PMC7613035; doi:10.1017/S1368980021002457)
Supplement: Supplementary file 1 [file S1368980021002457sup001.docx]

**Supplementary Table 1. Health Surveys and Census years that contributed to the Socioeconomic Development Index**

| **Country** | | | | | | | | |
| --- | --- | --- | --- | --- | --- | --- | --- | --- |
|  | **Brazil** | **Chile** | **Colombia** | **Costa Rica** | **El Salvador** | **Guatemala** | **Mexico** | **Peru** |
| **Number of cities included in this analysis** | 27 | 29 | 33 | 1 | 3 | 1 | 59 | 23 |
| **Number of participants included in this analysis (including women and men)** | 25 416 | 2 465 | 5 749 | 1 164 | 1 495 | 1 038 | 4 066 | 11 793 |
| **Cluster average size (min, max)** | 941 (494, 2 365) | 218 (25, 703) | 224 (63, 964) | 1 164 (NA) | 498 (161, 991) | 1 038 (NA) | 133 (23, 955) | 554 (155, 3 238) |
| **Health survey and year** | Pesquisa  Nacional de Saúde, PNS  (National Health Survey)  2013 | Encuesta Nacional de Salud (National Health Survey)  2010 | Encuesta Nacional de Salud (National Health Survey)  2007 | Encuesta Multi-  nacional de Diabetes y Factores de  Riesgo, CAMDI  (Central America Diabetes Initiative Survey)  2005 | Encuesta  Nacional de Enfermedades Crónicas no transmisibles en Población Adulta de El  Salvador ENECA (National Survey of Non-  communicable Chronic  Diseases in the Adult  Population of El Salvador)  2014-2015 | Encuesta Multi-  nacional de Diabetes y Factores de  Riesgo, CAMDI  (Central America Diabetes Initiative Survey)  2002 | Encuesta  Nacional de Salud y  Nutrición, ENSANUT  (National Survey for  Health and Nutrition)  2016 | Encuesta  Nacional de Demografia  y Salud, ENDESA (National Survey of Demographics and Health)  2016 |
| **Health surveys’ sample characteristics** | Age: All ages  Total N: 64 308 adults 18+ years  N in SALURBAL: 40 703 adults 18+ years | Age: ≥15 years  Total N: 5 293  N in SALURBAL: 3 140 | Age: 0 - 69 years  Total N: 102 677 (41 281 adults 18-69 years)  N in SALURBAL: 43 182 (18,783 adults 18-69 years | Age: ≥20 years  Total N: 1 427  N in SALURBAL: 1 427 | Age: ≥20 years  Total N: 4 817  N in SALURBAL: 1 546 | Age: ≥20 years  Total N: 1 397  N in SALURBAL: 1 397 | Age: all ages  Total N: 29 797 adults 18+ years    N in SALURBAL: 14 618 adults 18+ years | Age: All ages  Total N: 122 368 (adults 18+ years N=32158)  N in SALURBAL:  11 929 |
| **Health surveys’ sampling strategy** | Multistage [census tracts or groups of census tracts; households; person 18 years or older]  Stratified [capital city, metropolitan region, or integrated economic development region, then rest of municipalities; Urban/rural; total household income | Multistage [Comunas; Segments within comunas; household; person 15 years or older]  Stratified [urban/rural with three groups of population sizes] | Multistage [Municipalities or combination of municipalities if small; Manzanas; household; person adults 18-69 and all children 17 and under]  Stratified [region; urbanization of municipal seats; urban/rural municipal  population; unsatisfied basic needs] | Multistage [Census segments; groups of house-holds (compactos); Per-sons within three age groups (1 selected from 20-39 years, 1 selected from 40-64 years, all selected from >=65 years)] | Two-stage [Segmento censal, groups of dwell-ings (compacto); all household members 20 years and older] | Multistage [Segmento censal, groups of dwellings (compacto); all household members 20 years and older] | Multistage [AGEB; Manzana (urban) or pseudo-manzanas within localidades (rural); House-holds; 1 person within each of the groups (0-4 years, 5-9 years, 10-19 years, 20 years and older, recent medical ser-vice user)]  Stratified [socioeconomic status of AGEB at the state level] | Multistage [Con-glomerado (set of census blocks – urban) or Empadronamiento (set of households – rural); Households; One person within each of the groups (>15 years, females 15-49 years, children <5 years, children <12 years)]    Stratified [Department; Urban/Rural] |
| **Health surveys’ representation** | Regions (5) States or federation units (27), state capitals (27), urban and rural, metropolitan areas and development integrated areas | National, Regions (15), urban/rural | Region, department, sub-region, urban area of municipal capitals, urban/rural, by poverty level | Metropolitan San Jose | National, Urban National, Rural | Villa Nueva Municipio | National, state, metropolitan areas, urban/rural, high/low SES | National, Urban National, Rural National, Natural Region: Lima Metropolitan area, coast/mountain/jungle |
| **Year of census** | 2010 | 2017 | 2005 | 2011 | 2007 | 2002 | 2010 | 2017 |

NA= Not applicable.

**Supplementary Table 2. City-level Socioeconomic Development Index’s components in 176 Latin American cities by country**

|  | **Country** | | | | | | | |
| --- | --- | --- | --- | --- | --- | --- | --- | --- |
| **Index components** | **Brazil**  **% (95 % CI)** | **Chile**  **% (95 % CI)** | **Colombia**  **% (95 % CI)** | **Costa Rica**  **% (95 % CI)** | **El Salvador**  **% (95 % CI)** | **Guatemala**  **% (95 % CI)** | **Mexico**  **% (95 % CI)** | **Peru**  **% (95 % CI)** |
| Water access (% households with piped water) | 92.7 (68.4, 99.1) | 97.8 (85.2, 99.2) | 84.6 (34.7, 96.0) | 99.4 (99.3, 99.5) | 79.5 (66.3, 84.1) | 75.5 (75.4, 75.6) | 82.3 (39.6, 96.1) | 72.2 (36.4, 84.6) |
| Sanitation (% households with access to a municipal sewage network) | 54.4 (7.8, 87.9) | 96.4 (84.5, 98.9) | 85.8 (11.5, 95.9) | 34.9 (39.8, 34.9) | 68.3 (50.4, 76.7) | 69.9 (69.8, 69.9) | 84.6 (8.1, 96.6) | 74.6 (35.7, 92.7) |
| Durable walls (% of dwellings with exterior walls mostly made of brick, stone, concrete, cement and/or similar materials) | 92.6 (57.2, 99.8) | 68.1 (13.9, 89.7) | 90.9 (60.5, 97.4) | 73.5 (73.4, 73.6) | 85.6 (71.8, 90.1) | 83.3 (83.2, 83.4) | 93.6 (68.7, 99.2) | 68.1 (23.2, 97.4) |
| Overcrowding (% households with more than three people per room) | 4.7 (1.3, 11.6) | 3.9 (1.7, 6.9) | 5.5 (2.0, 18.6) | 0.6 (0.5, 0.7) | 12.9 (12.3, 14.6) | 17.2 (17.1, 17.4) | 8.1 (2.6, 16.6) | 10.0 (3.0, 23.8) |
| Education (% population with at least completed primary education among those aged 25 or above) | 70.7 (63.0, 78.1) | 87.4 (74.2, 92.9) | 77.8 (66.5, 85.7) | 86.2 (86.1, 86.3) | 65.1 (51.2, 69.9) | 64.9 (64.8, 64.9) | 82.1 (61.9, 88.2) | 81.0 (66.1, 88.0) |

**Supplementary Figure 1. Box plots of the city-level Socioeconomic Development Index in 176 Latin American cities by country**

BR – Brazil; CL – Chile; CO – Colombia; CR – Costa Rica; GT – Guatemala; MX – Mexico; PE – Peru; SV – El Salvador

**Supplementary Figure 2. Variability of BMI in women in 176 Latin American cities by country**

BR – Brazil; CL – Chile; CO – Colombia; CR – Costa Rica; GT – Guatemala; MX – Mexico; PE – Peru; SV – El Salvador

**Supplementary Figure 3. Variability of BMI in men in 176 Latin American cities by country**

BR – Brazil; CL – Chile; CO – Colombia; CR – Costa Rica; GT – Guatemala; MX – Mexico; PE – Peru; SV – El Salvador
